# Parallel-Channel Electrotaxis and Neuron Screening of *Caenorhabditis elegans*

**DOI:** 10.3390/mi11080756

**Published:** 2020-08-04

**Authors:** Khaled Youssef, Daphne Archonta, Terrance Kubiseski, Anurag Tandon, Pouya Rezai

**Affiliations:** 1 Department of Mechanical Engineering, York University, Toronto, ON M3J 1P3, Canada; kyoussef@yorku.ca (K.Y.); daphnea@my.yorku.ca (D.A.); 2 Department of Biology, York University, Toronto, ON M3J 1P3, Canada; tkubises@yorku.ca; 3 Tanz Center for Research in Neurodegenerative Diseases, Toronto, ON M5T 0S8, Canada; a.tandon@utoronto.ca

**Keywords:** *C. elegans*, microfluidics, electrotaxis, Parkinson’s disease

## Abstract

In this paper, we report a novel microfluidic method to conduct a *Caenorhabditis elegans* electrotaxis movement assay and neuronal imaging on up to 16 worms in parallel. *C. elegans* is a model organism for neurodegenerative disease and movement disorders such as Parkinson’s disease (PD), and for screening chemicals that alleviate protein aggregation, neuronal death, and movement impairment in PD. Electrotaxis of *C. elegans* in microfluidic channels has led to the development of neurobehavioral screening platforms, but enhancing the throughput of the electrotactic behavioral assay has remained a challenge. Our device consisted of a hierarchy of tree-like channels for worm loading into 16 parallel electrotaxis screening channels with equivalent electric fields. Tapered channels at the ends of electrotaxis channels were used for worm immobilization and fluorescent imaging of neurons. Parallel electrotaxis of worms was first validated against established single-worm electrotaxis phenotypes. Then, mutant screening was demonstrated using the NL5901 strain, carrying human α-synuclein in the muscle cells, by showing the associated electrotaxis defects in the average speed, body bend frequency (BBF), and electrotaxis time index (ETI). Moreover, chemical screening of a PD worm model was shown by exposing the BZ555 strain, expressing green fluorescence protein (GFP) in the dopaminergic neurons (DNs), to 6-hydroxydopamine neurotoxin. The neurotoxin-treated worms exhibited a reduction in electrotaxis swimming speed, BBF, ETI, and DNs fluorescence intensity. We envision our technique to be used widely in *C. elegans*-based movement disorder assays to accelerate behavioral and cellular phenotypic investigations.

## 1. Introduction

High-throughput screening (HTS) is a crucial drug discovery process that aims to test large compound libraries on a specific target in a sensitive, fast, and cost-effective manner [1]. Typically, preliminary hits are achieved by using in-vitro cell-based assays. The positive hits are then tested on whole-animal mammalian models to evaluate the chemical potency and toxicity before preliminary clinical trials [2]. Very commonly, these compounds are found to be impractical on whole animals due to the drug toxicity, metabolism complications, or poor target engagement, thereby rendering the process expensive and tedious [2]. Model organisms such as *Caenorhabditis elegans* (*C. elegans*) [1,3,4], *Drosophila melanogaster* [5,6], and *Danio rerio* [7] have shown promising outcomes to fill the gap between in-vitro cell-based and in-vivo whole-animal studies.

*C. elegans* is a free-living worm and a promising model for studying human diseases due to its genetic homology with humans, small size, short life cycle, cost-effective maintenance, fecundity, and whole-life body transparency for fluorescent imaging of neuron and muscle cells [8,9]. Moreover, *C. elegans* continues to be of importance in drug discovery due to a fully sequenced genome, genetic tractability, and many other experimental advantages [8,9]. *C. elegans* behavioral phenotypes such as mobility, body morphology, pharyngeal pumping, brood size, and development, along with in-vivo fluorescently labeled cells, have been quantified for drug efficacy testing [10,11,12,13]. For instance, *C. elegans* share various gene orthologues for many of the neurological disorders. Therefore, they have been exploited extensively as models for neurodegenerative diseases (NDs), such as Parkinson’s disease (PD) [14,15,16,17], Alzheimer’s disease (AD) [18], and Huntington’s disease (HD) [10]. Various mutants have been created to help reveal the causes underpinning these NDs and to identify novel neuroprotective compounds [19].

Microfluidics have contributed to *C. elegans*-based ND research by offering various manipulation and screening platforms. The prevision offered by microfluidics in delivering external stimuli and maintaining highly controllable test conditions has facilitated its use in evoking the worms’ neurobehavioral phenotypes for chemical screening. For example, Ma et al. [20] and Shi et al. [21] investigated the effects of 1-methyl-4-phenylpyridinium (MPP+) and 6-hydroxydopamine (6-OHDA), respectively, on worms’ mobility and neurodegeneration rate to study worm models of PD using microfluidic platforms. Recently, Mondal et al. [22] invented a novel drug screening platform based on the worms’ fluorescently tagged neurons to screen for various drugs in a short time. The chip was designed in a 96-well plate format to fit within an automated liquid handling system, and a worm model of HD was used to screen for positive hits out of 1000 FDA-approved compounds.

In addition to the natural behaviors of the worm investigated in the papers above, induced responses by different stimuli, such as chemicals, light, temperatures, magnetic fields, and electric fields have also attracted attention [8,23,24,25,26]. For instance, Salam et al. [27] exploited the innate response of *C. elegans* towards the cathode under the effect of a direct current (DC) electric field in a microchannel, termed electrotaxis [28], as an on-demand method for drug testing. Various PD-related neurotoxins were utilized to validate the use of electrotaxis in assessing neurobehavioral processes. To enhance the speed of this technique, Li et al. [29] developed an automated system to achieve a throughput of 20 worms/h in a single-channel single-worm device and validated the system using a worm model of PD. Worms’ electrotaxis behavior on open-surface substrates has been shown to be relatively complex due to electric field nonuniformity and multidirectional movement of worms, but in the above microfluidic approaches, microchannels have provided uniform and consistent stimulus exposure and movement pathways to guide worms directionally for easy phenotypic quantification.

Up until now, electrotaxis assays on freely moving worms have been done on a single worm at a time, and no on-chip imaging along with electrotaxis screening has been reported. Testing of multiple worms to enhance the throughput of electrotaxis screening and simultaneous neuron imaging to correlate movement malfunctions with neuron and muscle degeneration, preferably at single animal resolution, is still needed. To address these gaps, we report a simple and easy-to-use microfluidic electrotaxis-based chip to investigate the behavior and neuron degeneration of 16 worms in parallel. In this context, we showed the applicability of our device for genetic, chemical, and neuronal screening after validating it against the single-worm electrotaxis assay.

## 2. Materials and Methods

### 2.1. Chemicals and Materials

For the lithography procedures, a set of 4-inch diameter and 500–550 μm thick silicon (Si) wafers was obtained from Wafer World Incorporation (West Palm Beach, FL, USA). SU8 developer and the negative photoresist SU8-2035 were procured from MicroChem Corporation (Newton, MA, USA). Polydimethylsiloxane (PDMS) was ordered from Dow corning Corporation (Auburn, MI, USA).

All other chemicals were ordered from Sigma-Aldrich (St. Louis, MO, USA). Typically, *C. elegans* M9 buffer was prepared by autoclaving a 1 L solution of 3 g of KH_2_PO_4_, 6 g Na_2_HPO_4_, and 5 g NaCl in distilled H_2_O, followed by the addition of 1 ml of 1 M MgSO_4_. *C. elegans’* food source of *Escherichia coli (E. coli)* strain OP50 was prepared in L-broth, a bacterial food source. L-broth was obtained by autoclaving a 1 L mixture of 10 g of Bacto-tryptone, 5 g of Bacto-yeast, and 5 g of NaCl in distilled H_2_O. For neurodegeneration, 6-OHDA (636-00-0, Sigma-Aldrich), a known neurotoxin for degenerating the dopaminergic neurons, was used by obtaining a 10 mM stock solution using 5 mg of 6-OHDA in 2 mL of autoclaved M9. 6-OHDA solution was prepared in a dark room and stored at −20 ${}^{\circ }$C.

### 2.2. C. elegans Strains, Maintenance, Synchronization, and Chemical Exposure

Wild-type N2, BZ555, and NL5901 strains (obtained from the *Caenorhabditis* Genetics Center (University of Minnesota, Minneapolis, MN, USA)) (Table 1) were grown on standard nematode-growth media agar plates seeded with OP50 as a food source at 25 ${}^{\circ }$C. For all assays, worms were synchronized by Alkaline hypochlorite treatment, as previously described. [30] Briefly, gravid adult hermaphrodites were washed off the plate using M9 buffer and centrifuged for bacterial removal. Then, the worms’ pellet was treated with a commercial bleach-based solution (1 mL of commercial bleach, 125 μL of NaOH, and 3.875 mL of double-distilled water) for egg-extraction. The extracted eggs were allowed to hatch into L1 larve overnight in 1 mL of M9 buffer. In the following day, the hatched larvae (L1 stage) were treated with 250 μM of 6-OHDA (975 μL M9 and 25 μL 6-OHDA from our prepared 10 mM stock solution) in a dark room for 1 h [27]. The control batches were only treated with M9 for 1 h in the darkroom to maintain the same test conditions. The worms were incubated for 40 h at 25 ${}^{\circ }$C to be tested at the young adult stage.

### 2.3. Experimental Setup and Device Design

**Figure 1:**
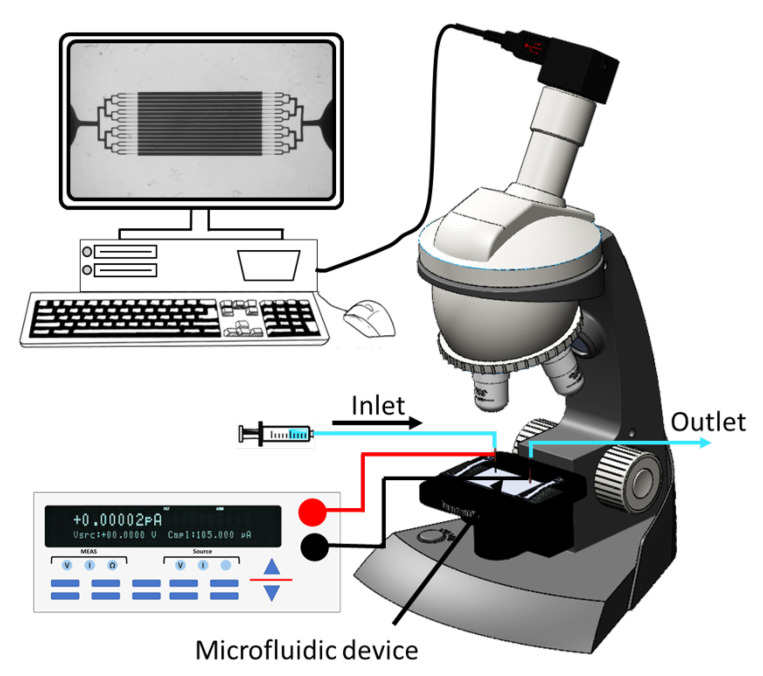
Sketch of our experimental setup consisting of our microfluidic device, a microscope, a camera, a sourcemeter, and a computer.

**Figure 2:**
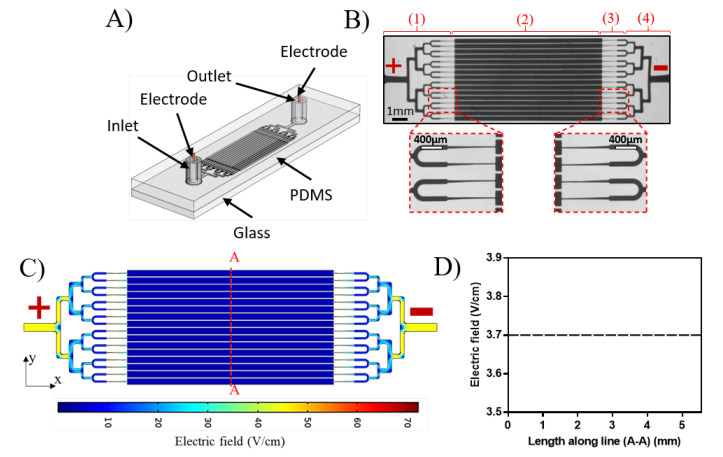
(**A**) Schematic of the parallel electrotaxis microfluidic chip (3 cm × 1.5 cm) consisting of one inlet and one outlet that are connected by four modules shown in (**B**): (1) worm loading and distribution channels, (2) 16 parallel electrotaxis screening channels, (3) tapered channels for worm immobilization and imaging, and (4) unloading channels. (**C**) Electric field distribution throughout the chip simulated using COMSOL by applying 50 V to achieve a constant electric field of 3.7 V/cm in electrotaxis screening channels (**D**).

The experimental setup used to perform this study is illustrated in Figure 1. It consisted of a microfluidic device (Figure 2A) with two end electrodes connected to a Keithley 2410 DC sourcemeter (Keithley Instruments Inc., Austin, TX, USA). Imaging of worms inside the device was done by an upright microscope (Leica MZ10F fluorescence microscope, Leica, Wetzlar, Germany). The mono-layer device was fabricated from (PDMS) using conventional soft lithography [33,34] and irreversible bonding to a glass slide using O_2_ plasma [23] (see Appendix A for details). Our device consisted of four-channel sections, each 60 μm-thick, as shown in Figure 2B, i.e., (1) branching channels for worm loading and distribution; (2) 16 parallel 300 μm-wide electrotaxis screening channels; (3) tapering channels, from 40 to 20 μm, for worm immobilization and imaging; and (4) branching channels for unloading the worms. The electrotaxis screening channels were designed according to the results of Rezai et al. [28] to allow proper worm swimming and turning (see Appendix C for details). Electrodes were installed in inlet and outlet reservoirs for electric field stimulation.

The critical design criteria of our chip were to smoothly load the worms and provide a constant electric field throughout all the 16 screening channels for electrotaxis studies. This was achieved by embracing a loading technique inspired by Hulme et al. [35] through a hierarchy of channels that helped maintaining equal hydrodynamic and electrical resistances for each path. Hydrodynamic resistance determines the path each worm follows at each bifurcation in the network. An occupied path will lead the next worm to be loaded into another vacant channel. Constant channel dimensions at each bifurcation were used to maintain the same pressure and voltage drop up to the electrotaxis screening channels using Hagen–Poiseuille’s and Ohm’s laws [36]. The pressure and voltage drops were defined by Equations (1) and (2), respectively.

(1) $$▵P=R_{f}Q,\;R_{f}=\frac{128\mu l}{\pi D^{4}}$$

(2) $$▵V=R_{e}I,\;R_{e}=\frac{\rho l}{A}$$

where Q is the flow rate, $R_{f}$ is the fluid flow resistance, D is the channel hydraulic diameter, l is the channel length, μ is the fluid dynamic viscosity, A is the cross-sectional area, $R_{e}$ is the electrical resistance, I is the electric current, and ρ is the electric resistivity.

Two-dimensional steady-state COMSOL simulations (accessed via CMC Microsystems) were conducted to estimate the electric field across the channels (see Appendix B for details). Fluid electric conductivity was obtained experimentally using a 3 cm-long and 300 μm-wide channel. Using custom-written MATLAB code, various voltages were applied, and the electric current across the channel was obtained to calculate the channel’s electric resistance. Using the electric resistance and the known channel dimensions in Equation (2), the fluid electric conductivity was found to be approximately 1.6 siemens/m. Figure 2C shows the electric field distribution across the microfluidic chip at a constant electric voltage of 5 0V. No electric field variation was observed along line A-A in Figure 2C, and the electric field (EF) was 3.7 V/cm across all the channels (Figure 2D). The obtained EF satisfied the required EF range of 2–4 V/cm needed for young adult *C. elegans’* electrotaxis [28].

### 2.4. Experimental Methodology

**Figure 3:**
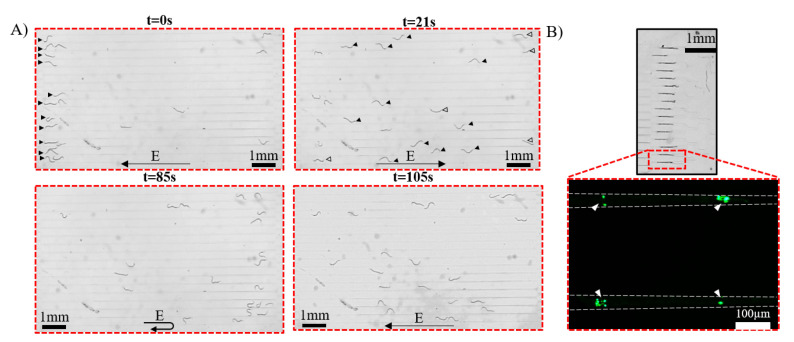
(**A**) Time-lapse images showing electrotaxis of N2 worms in parallel channels after exposure to 250 μM 6-OHDA. Black arrows indicate worms responding towards the cathode, while empty arrows show worms with different phenotypes, such as uncoordinated movement and sudden freezing due to 6-OHDA exposure (EF = 3.7 V/cm). (**B**) Worms trapped in the tapered channels, with a zoom-in on two immobilized BZ555 worms, fluorescently imaged in a healthy state. Arrowheads are showing the dopaminergic neurons (DNs).

Young adult worms were loaded into the microchannel using a syringe and pushed slowly until all the tapered entrance channels were occupied by worms (Supplementary Materials Video S1). Next, the worms were manually pressure-pulsed and placed in the screening channels (Figure 3A). In 5 trials, N = 12 ± 3 worms were successfully loaded into channels.

To permit free swimming, the flow rate was brought to zero by releasing the loading pressure and letting the worms stabilize in the channel. A constant DC electric field of 3.7 V/cm was applied in the screening channels, which initiated the worms’ movement towards the cathode for 10 mm (Figure 3A shown for 6-OHDA exposed worms). Once the worms reached the end of the electrotaxis channel, the electric field was reversed, thereby triggering the worms to turn and swim in the opposite direction (Supplementary Materials Video S1). This was repeated twice, and behavioral phenotypes were determined and reported as described below.

The electrotaxis swimming speed and body bend frequency (BBF) were quantified using the automated Worm Tracker plugin in ImageJ software [37]. The electrotaxis turning time (ETT) and electrotaxis time index (ETI) [23,27] were calculated for all conditions and averaged over the three electric field exposure cycles. ETT is the time at which the worm successfully performed a complete turn after an electric field reversal and started to swim towards the cathode. ETI is the ratio between the actual swimming time towards the cathode to the total time of the experiment. It was defined to account for the intermittent stops and reversals happening during the movement towards the cathode. For fluorescent imaging, the worms were aspirated into the trapping channels for imaging (Figure 3B and Video V1).

### 2.5. Data Analysis

#### 2.5.1. Quantification of Neuron Degeneration

Neurodegeneration was assayed by simultaneously immobilizing the worms using parallel tapered channels (Figure 3B). The worms were imaged using fluorescent microscopy. The images were quantified in terms of their fluorescence intensity using ImageJ software (national institute of Health, NIH, Bethesda, MD, USA) [37]. Briefly, ImageJ software was used to subtract the background of each image using the built-in rolling ball algorithm [38] and we calculated the mean fluorescence intensity (MFI) of the entire image. The drug-treated worms’ MFIs were normalized with the control experiments using Microsoft Excel (Microsoft Corp., Redmond, WA, USA).

#### 2.5.2. Statistical Analysis

All the results are presented as mean ± standard error of the mean (SEM), while the difference among the two populations was compared using the Mann–Whitney test. The data were deemed significantly different at a *p*-value of less than 0.05. The star-based notation was used to identify the significance level as follows: * for *p* < 0.05, ** for *p* < 0.01, *** for *p* < 0.001, and **** for *p* < 0.0001.

## 3. Results and Discussion

**Figure 4:**
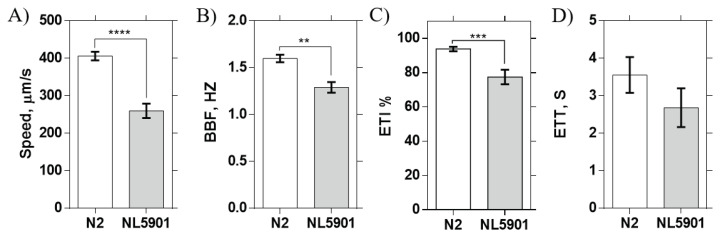
Application of the microfluidic device to mutant screening using NL5901 strain expressing α-syn (N = 19/21 responders) at EF = 3.7V/cm. (**A**) Worm speed, (**B**) body bend frequency (BBF), (**C**) electrotaxis time index (ETI) of responder worms, and (**D**) electrotaxis turning time (ETT). Error bars are SEM; *: *p* < 0.05; **: *p* < 0.01; ***: *p* < 0.001; ****: *p* < 0.0001.

The performance of our device was first confirmed by investigating the electrotaxis responses of wild type worms. As shown in Figure 4, N2 worms showed an average speed of 406 ± 36 μm/s, turning time of 3.5 ± 0.48 s, and BBF of 1.6 ± 0.125 Hz. These results matched the previously published electrotaxis results in a single channel device with an electric field of 4 V/cm [29] (see Appendix C for details), highlighting the applicability of our method for multi-worm electrotaxis screening. N = 12 ± 3 worms could be successfully loaded and tested in our device, with the assay taking on average six minutes for all worms. This significantly reduced the electrotaxis test time for each worm from 3–4 min [28] to about 30 s in our parallel-channel device.

### 3.1. α-syn Aggregation Effect on C. elegans’ Electrotaxis

Parallel electrotaxis was then used to perform mutant screening. We conducted experiments on NL5901 worms expressing α-syn in muscle cells. α-syn is a protein that aggregates to create insoluble fibrils that coalesce in cytoplasmic inclusions called Lewy bodies, a pathological hallmark of PD [17,39]. Transgenic worm lines expressing α-syn have been reported to show reduced lifespans, motility, and pharyngeal pumping rates. [40] Thus, we aimed to examine whether α-syn overexpression in muscles alters stimuli-evoked behavioral responses, such as electrotaxis, to identify genetically-induced movement deficits. Figure 4 shows that α-syn overexpression induced significant decreases in the worms’ average speed, BBF, and ETI, whereas no effect on the ETT was observed. These findings implied that α-syn aggregation in muscles affect the worms’ response to the electric field, causing a decrease in worm motility and difficulty for the worm to maintain continuous swimming towards the cathode (as per low ETI). In the future, it would be interesting to test a strain with α-syn overexpression in DNs to interrogate the behavioral effect of protein aggregation inside the neurons.

### 3.2. Chemical Screening Using a PD-Related Neurotoxin

**Figure 5:**
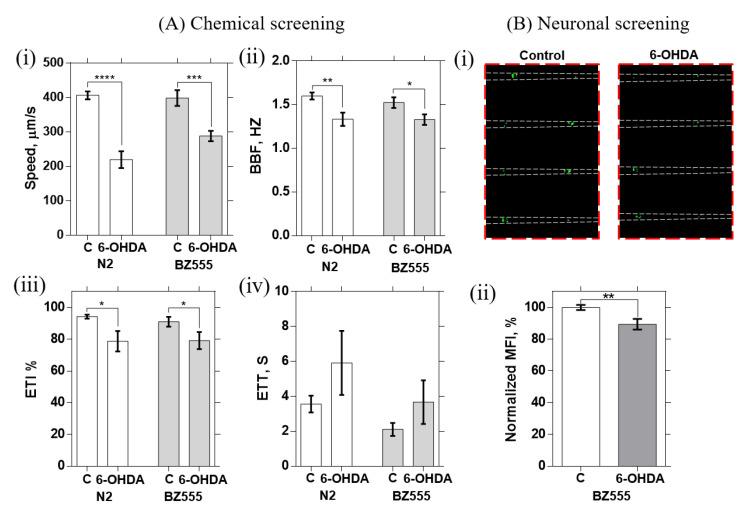
Application of the microfluidic device to chemical and neural screening at EF = 3.7 V/cm using N2 (wild-type) (N = 16/17 responders for control and N = 27/29 responders for exposed worms) and BZ555 strain (N = 19/20 responders for control and N = 24/29 responders for exposed worms) exposed to 250 μM 6-OHDA (controls are shown by “C”). (i) Worm speed, (ii) body bend frequency (BBF), (iii) rlectrotaxis turning time (ETT), and (iv) electrotaxis time index (ETI). Error bars are SEM; *: *p* < 0.05; **: *p* < 0.01; ***: *p* < 0.001; ****: *p* < 0.0001.

To further demonstrate the application of our device in chemical screening, worms’ electrotaxis response after exposure to 6-OHDA was studied. 6-OHDA is a neurotoxin that has been reported to induce PD-like symptoms by selectively degenerating the DNs. [19] DNs take up 6-OHDA through the dopamine transporter DAT-1, which leads to oxidative stresses and cell death. [41] Changes in dopamine levels will result in various neurological disorders, including PD. In this test, N2 and the transgenic strain BZ555, which expressed GFP in the DNs, were used to screen for mobility defects upon electric field stimulation (Figure 5A) and neurodegeneration upon exposure to 250 μM 6-OHDA. Typically, the untreated N2 and BZ555 worms exhibited normal swimming speed, BBF, and ETT, and high ETI, attributed to their healthy state, whereas the 6-OHDA treated worms showed a slower response in terms of swimming speed, BBF, and ETI. After electrotaxis screening of BZ555 worms, the DNs were fluorescently imaged in the tapered channels (Figure 3B and Figure 5B-i), and their MFI was quantified (Figure 5B-ii). The untreated worms’ DNs were intact with strong GFP expression, contrary to the treated worms, which showed a reduction in the MFI due to the partial loss of DNs upon exposure to the neurotoxin.

## 4. Conclusions

In conclusion, this work demonstrated an easy to operate, simple to fabricate, and reusable microfluidic device for the analysis of the electrotaxis responses of multiple worms at single animal resolution. We showed that this device can be used in a wide range of *C. elegans* assays wherein movement and cellular phenotypes need to be investigated on large groups of specimens, such as neurodegenerative disease studies and chemical screening. Moreover, considering the limitations associated with microfluid devices’ integrability in biological laboratories, we developed our device to be simple to use by an end-user with the aid of syringes and a power supply; it also increased the number of worms that can be tested simultaneously, achieving at least nine worms every 5 min, which has not been achieved previously for electrically induced-behavioral assays even with automated systems. We envision that although automating this system will add complexity to it, it could help the throughput to reach to more than 100 worms per hour in the future.

## Figures and Tables

**Table 1:** *Caenorhabditis elegans* strains used in this study.

Strain	Genotype	Description	Ref.
N2	WT Bristol	Wild type	
NL5901	*pkIs2386 [unc-54p:: α-syn::YFP+unc-119(+)]*	α-syn YFP expression in muscle cells	[31]
BZ555	*egIs1 [dat-1p::GFP]*	GFP expression in DNs	[32]

**Figure A1:**
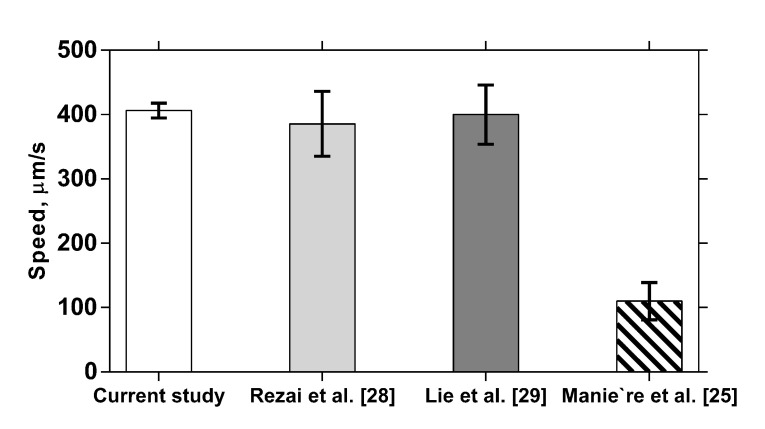
Comparison of the swimming speeds obtained using the multi-worm assay in the current study, and the single worm electrotaxis assays from Rezai et al. [28] and Liu et al. [29], plus the off-chip crawling electrotaxis speed on agar surfaces from Manie ‘re et al. [25].

## Supplementary Materials

The following are available at https://www.mdpi.com/2072-666X/11/8/756/s1, Video S1: Worm loading, electrotaxis testing, and the neuron imaging procedure in the microfluidic device.

## Appendix A. Microfluidic Chip Fabrication

The conventional photolithography [34] technique was used to fabricate a 60 μm-thick monolayer SU8-mold. A pre-treated 4 inch Si wafer was used as a substrate. Four milliliters of the negative SU-8 2035 photoresist was poured and pre-spun at 500 rpm for five seconds. Then, to achieve a thickness of 60 μm, the spinning speed increased to 1700 rpm for 30 s, followed by soft-backing at 65 ${}^{\circ }$C for 1.5 min and 95 ${}^{\circ }$C for 7.5 min. A computer-aided design was sketched and printed as a 25,000 DPI transparency photomask (CAD∖Art Services Inc., USA). UV-KUB2 (KOLE, France) was used to expose the Si wafer, using the transparency mask, to ultraviolet light at 365 nm with a power of 10 mW/cm${}^{2}$ for 18 seconds, followed by post-backing at 65 ${}^{\circ }$C for one minute and 95 ${}^{\circ }$C for six minutes. Finally, the wafer was rinsed with SU8-developer, followed by IPA, and hard-baked at 200 ${}^{\circ }$C for 10 min. A Bruker optical profilometer was used (Bruker Optics, USA) to confirm the wafer’s thickness.

The PDMS device was fabricated using the standard soft lithography technique. [33] The inlet and outlet were prepared by attaching a piece of Masterflex tubing (L/S 14 size, Gelsenkirchen, Germany) over the reservoirs on the master mold. A mixture of PDMS elastomer base and curing agent in the ratio of 10:1 was prepared, de-gasified, poured over the Si wafer, and left to cure for two hours at 80 ${}^{\circ }$C. The cured PDMS was peeled off the wafer and bonded irreversibly to glass using oxygen plasma (PDC-001-HP Harrick Plasma, USA) at 870 mTorr pressure and 30 W for 30 s. The electric wires were connected to the inlet and outlet tubes, for electric field stimulation, by punching through the PDMS and sealing with liquid PDMS.

## Appendix B. Numerical Simulation of the Electric Field

The commercial software COMSOL Multiphysics® was used to predict the electric field distribution in the microfluidic device. Two-dimensional (2D) numerical simulations were conducted to solve Ohm’s law using the steady-state direct-current electric module to obtain the electric field within a conductive media. SOLIDWORKS® software was utilized to generate the computational domain (Figure 2 of the paper), which was then imported into the COMSOL Multiphysics software for the mesh generation and boundary conditioning. Three boundary condition types were adopted: an electric potential of 50V at one of the end reservoirs, ground for the other end, and electric insulation for all other boundaries. M9 was used as the conductive media, and its electric conductivity was found experimentally to be approximately 1.6 siemens/meter. The number of meshes was set to be approximately 1.5 × 10${}^{6}$ after conducting a mesh independency study. Figure 2C of the main paper illustrated the electric field distribution across the microfluidic device, showing that the electric field is constant across each electrotaxis channel and consistent across other channels.

## Appendix C. Comparison of Multi-Worm and Single Worm Electrotaxis Assay

In the conventional electrotaxis assay, a single worm can be stimulated to swim towards the negative pole, and some behavioral phenotypes, including speed, body bend frequency (BBF), electrotaxis time index (ETI), and electrotaxis turning time (ETT) are quantified. Here, we present a multi-worm electrotaxis assay for 16 worms in parallel that can provide information for the same phenotypes and image the worms fluorescently using a tapered channel immobilization technique, which has not been achieved previously in the single worm electrotaxis assay.

*C. elegans* electrotaxis has been studied in terms of crawling on open agar gel surfaces or swimming in media inside microfluidic devices. For instance, in 2007, Gabel et al. [24] studied the electrotaxis behavior of *C. elegans* on open gel surfaces using two stereotyped maneuvers, but movement speed was not reported. Then, Manière et al. [25] studied the same behavior in open gel surfaces and reported a crawling speed of 110 ± 50 μm/s towards the negative pole. Crawling speed is expected to be lower than swimming speed. Using microfluidic devices, different groups have studied electrotaxis swimming and established that the 300 μm channel width is the optimum dimension for obtaining a proper swimming speed [26,27,28,29]. Therefore, we adopted the same channel dimensions and replicated them to make 16 parallel channels for our experiments. In order to verify our technique, we compared the results obtained in our device for wild-type worms with the results obtained by other groups in terms of swimming speed. As shown in Figure A1, we obtained an average swimming speed of 406 ± 36 μm/s; compare that to the results obtained by Rezai et al. [28] and Liu et al. [29] in single channels. That supports the fact that the 300 μm channel is not affecting the worms’ movement and the worms in our device are responding normally to the EF as the single-worm electrotaxis assay. It should also be mentioned that the channel width in the range of 300–500 μm has been reported not to have any significant effect on the worms’ electrotaxis speed, while channels larger than 300 μm may cause some complexity in the response since the worms will gain freedom to move laterally in the channel and orient themselves at an angle with the electric field. It should also be mentioned that the differences between our results and those of Manière et al. [25] in Figure A1 may stem from differences between swimming and crawling, respectively.
